# Expression and characterization of hemagglutinin–neuraminidase protein from Newcastle disease virus in *Bacillus subtilis* WB800

**DOI:** 10.1186/s43141-022-00357-w

**Published:** 2022-05-24

**Authors:** Mohammadreza Shafaati, Masoud Ghorbani, Minoo Mahmoodi, Mostafa Ebadi, Reza Jalalirad

**Affiliations:** 1 Department of Cellular & Molecular Biology, Faculty of Basic Sciences, Hamedan Branch, Islamic Azad University, Hamedan, Iran; 2 Pasteur Institute of Iran, Production and Research Complex, Department of Research and Development, Kilometre 25 Karaj-Tehran Highway, Karaj, Alborz 31599 Iran; 3 Department of Biology, Faculty of Sciences, Damaghan Branch, Islamic Azad University, Damghan, Semnan Iran

**Keywords:** Newcastle disease virus, *Bacillus subtilis*, Secretory expression, Purification

## Abstract

**Background:**

Newcastle disease virus (NDV) belongs to the genus *Avaluvirus* and *Paramyxoviridae* family, and it can cause acute, highly contagious Newcastle disease in poultry. The two proteins, haemagglutinin neuraminidase (HN) and Fusion (F), are the main virulence factor of the virus and play an essential role in immunogenicity against the virus. In most paramyxoviruses, the F protein requires HN protein to fuse the membrane, and HN proteins substantially enhance the viruses’ fusion activity.

**Results:**

The present study describes the successful cloning and expression of HN protein from NDV in *Bacillus subtilis* WB800 using the modified shuttle vector pHT43. HN coding sequence was cloned into the pGet II vector. It was then subcloned into the PHT43 shuttle vector and transferred to *Escherichia coli* for replication. The recombinant plasmid was extracted from *E. coli* and used to transform *B. subtilis* by electroporation. After induction of recombinant *B. subtilis* by IPTG, total cell protein and the protein secreted into the media were analysed through a time course using SDS-PAGE. The expressed HN protein was purified using cation exchange chromatography followed by metal affinity chromatography, using the 6× His epitope introduced at the carboxyl terminus of the recombinant protein. The accuracy of the PHT43-HN construct was confirmed by sequencing and enzymatic digestion. SDS-PAGE results showed that the recombinant HN protein was successfully expressed and secreted into the medium. Moreover, the purified HN protein showed neuraminidase activity with characteristics similar to the indigenous HN NDV protein. *B. subtilis* is a free endotoxin host that could be a favourite prokaryotic platform for producing the recombinant HN protein.

**Conclusion:**

The establishment of this expression and purification system has allowed us to explore further the biochemical characteristics of HN protein and obtain material that could be suitable for a new production of NDV candidate vaccine with high immunogenicity.

## Background

The Newcastle disease virus (NDV) is a member of the *Orthoavulavirus* genus in the Paramyxoviridae family. The virus can cause the highly contagious Newcastle disease (ND) in poultry [1–4] resulting in a significant annual damage to the industry of poultry, especially in developing countries [2, 5, 6]. This enveloped virus has a single-stranded non-segmented RNA genome of negative sense [1, 2]. The NDV genome contains six major structural (3′-NP-P-M-F-HN-L-5′) and two minor proteins called W and V which are achieved through the process of RNA editing on the P gene by adding guanine nucleotides [7]. Viral replication, transcription, translation and protein processing all take place in the cytoplasm of the host cell, while virus particles are released through the plasma membrane through budding [8–10]. Haemagglutinin–neuraminidase (HN) and fusion (F) proteins play an essential role in immunogenicity against the virus and are the virus’ key virulence factors [11–14]. Two glycoproteins, F and HN, play essential roles in the assembly and development of envelop viruses and determining tropism in the host and tissues [14, 15]. The F protein induces fusion, while HN is responsible for binding [16–18]. HN glycoprotein has activities such as hemagglutination (HA) and neuraminidase (NA) and stimulates F protein activity [19]. The HN binding to the sialic acid receptor on the cell’s surface initiates membrane fusion by the F protein [20]. The HN of NDV is an integral membrane protein type II, which contains three main areas: a cytoplasmic tail in the N-terminals, a stalk membrane-proximal domain and a globular head domain the C-terminal [21–24]. The globular head in the C-terminal domain is where the receptor binding and enzymatic activity happen [25, 26], while it is also the site for neuraminidase activity. By removing sialic acid from the cell’s surface, the HN of NDV prevents the self-aggregation of the virus progenies during budding and helps with the release of the virus from the cell [27, 28].

*Bacillus subtilis* is an excellent host for expressing high levels of recombinant proteins [29, 30]. This non-pathogenic bacterium belongs to the group of GRAS bacteria [31]. The ability of the bacterium to release recombinants to the crop medium facilitates processes downstream and protein isolation and cleansing [31]. Using non-biassed codons affects the quality of large-scale protein production, which is one of the appealing features of this expression system [31].

The present study describes the successful cloning of the HN protein from the NDV and its expression in *B. subtilis WB800* using a modified pHT43 shuttle vector. In addition, the recombinant hemagglutinin–neuraminidase biochemical properties are characterized.

## Methods

### Virus, bacterial strains and plasmids

In this experimental study, the complete HN gene sequence (Accession No. AF07761.1) was obtained from the NCBI database. Razi Vaccine and Serum Research Institute of Iran provided the Iranian lentogenic LaSota strain of NDV grown in 10-day-old specific pathogen-free (SPF) embryonated chicken eggs. *B. subtilis WB800* (#PBS022-MoBiTec, Germany) was used as the expression host. Moreover, *Escherichia coli DH5α* (Invitrogen Inc.) was used for the amplification of recombinant plasmids. The pGet II plasmid (#CLo841-Sinaclon, Iran), containing an ampicillin and kanamycin-resistant gene, was used as the initial cloning/sequencing vector. The pHT43 shuttle vector (#PBS002-MoBiTec, Germany), containing ampicillin and chloramphenicol-resistant gene, was used to express the heterologous HN protein from the NDV.

### Media composition and culture conditions

Bacterial strains of *E. coli* and *B. subtilis* were grown aerobically at 37 °C and 200 rpm in a Luria–Bertani (LB) culture medium, containing 1% w/v of peptone, 0.5% w/v of yeast extract and 0.5% w/v of sodium chloride. LB medium will be supplemented with ampicillin amp 100 g/ml for *E. coli* or chloramphenicol 5 g/ml for *Bacillus*.

### RNA extraction and synthesis of the HN gene

According to the standard protocols, the lentogenic LaSota strain of NDV was propagated and harvested in SPF eggs [32]. Briefly, viral RNA was extracted from 200 μl supernatant of the harvested cell culture using a SinaPureTMViral (#EX6061-Sinaclon, Iran) commercial kit. Following the manufacturer’s guidelines, cDNA synthesis was performed using a ThermoScript™ RT-PCR (Invitrogen, USA). DNTPs (10 mM per base) and a special buffer for enzymatic reactions and an RNase inhibitor were used in the presence of specific primers (at 10 μM or 10 pmol/μl each for each primer). An RNAase/DNase-free micro-tubes (Extra gene, USA) were used in all experiments. Specific (forward and reverse) primers were designed using CLC Main Workbench 4.5 (QIAGEN Co.) to amplify the complete ORF of the HN gene of NDV (1734 bp). Moreover, the PCR reaction was performed using 5 μl of cDNA; 1 μl of the forward primer, i.e. 5′ GGA-TCC-ATG-GAC-CGC-GCC-GTT-AG 3′; 1 μl of the reverse primer, i.e. 5′ TCT-AGA-CTA-GCC-AGA-CCT-GGC-TTC-TC 3′ (underlined nucleotides correspond to BamHI and XbaI sites, respectively); 10 μl of the reaction buffer (containing Tris-Cl at the pH of 8.3 and 50 mM of KCl); 1.5 mM of the MgCl2 buffer; 10 mM of dNTPs; and one unit of high-fidelity PCR Enzyme Mix (Genetbio-Korea) in a total volume of 20 μl in a thermocycler device (Biorad, USA) below the subsequent conditions: 5 min at 95 °C, followed by 30 cycles at 95 °C for 30 s, 60 °C for 30 s, and 72 °C for 80 s, with a final extension step at 72 °C for 7 min. The PCR products were then analysed on 1% (w/v) agarose gel and stained with a safe view (Kiagene, IRI) [33, 34].

### Cloning and construction of the expression plasmid containing the HN gene

The RT-PCR product was extracted from low melting agarose gel using DNA extraction kit (Vivantis-Korea). Subsequently, it was cloned in the pGet II-T/A cloning vector (SMOBio-TW, #CV1100) to obtain the recombinant pGet-HN plasmid. After amplification in *E. coli* DH5α, the target gene was subjected to automatic one-directed sequencing using forward and reverse primers, described previously for the HN gene amplification. Then, it was sub-cloned into the pHT43 donor plasmid (MoBiTec-GR, #PBS002C) using BamHI/XbaI sites to obtain the pHT43-HN recombinant expression vector. This vector uses a powerful promoter before the groESL *Bacillus subtilis* operon, induced by the Lock operator, and the induction is enabled by adding ITPG. An efficient Shine-Dalgarno (SD) sequence and a vector multiple cloning sites (BamH I, Xba I, AatII, SmaI) were inserted. This vector uses the amyQ signal peptide to obtain recombinant secretory proteins. The presence of the pHt43-HN recombinant vector was confirmed using PCR and enzymatic digestion analysis. After amplification in *E. coli* DH5α, the expression plasmids have been extracted and transferred into *B. subtilis WB800*.

### Protein structures prediction

The GOR4 (http://expasy.org/tools/gor4.html) and the Continuum Secondary Structure Predictor (http://pprowler.itee.uq.edu.au/sspred) servers were used to obtain the secondary structure of the HN protein. Secondary structure prediction defined each residue as an alpha helix, beta-sheet or random coil. The prediction of protein structure and production of 3D models of the protein was performed by the I-TASSER server (http://zhanglab.ccmb.med.umich.edu/I-TASSER) and Swiss modelserver (https://swissmodel.expasy.org). The three-dimensional structural quality of the HN protein was performed by online software, such as Uppsala Ramachandran Server (http://eds.bmc.uu.se/ramachan.html) and PROCHECK (http://swissmodel.expasy.org/workspace) were used for energy minimization.

### Expression and purification of the recombinant HN protein expression

In order to evaluate the expression in *B. subtilis WB800*, three bacterial groups were tested, i.e. (a) the expression of *B. subtilis WB800* carrying pHT43-HN recombinant plasmids, (b) the expression of *B. subtilis WB800* carrying pHT43 plasmids without the HN gene, and (c) the expression of *B. subtilis WB800* without plasmids. All three groups were cultivated overnight in 10-ml LB broth, containing 3 μg/ml of chloramphenicol (only the A and B groups) at 37 °C with centrifuging at 200 rpm. An aliquot (1.5 ml) of the overnight culture (1% v/v) was inoculated in the fermentation medium in a 50-ml flask containing chloramphenicol, followed by incubation for 8 h. When the culture reached OD_600_ of 0.7–0.8, it was split into two equal portions, followed by inducing expression with 1 mM of IPTG added to one portion (*t* = 0). The samples collected from the culture medium were 1 ml before the induction and 4 ml (3 ml per 2 h) after the induction. The process of precipitating the protein from the supernatant was performed by saturating it with NaCl, followed by centrifugation at 6000×*g* for 10 min at 4 °C. Subsequently, the protein’s expression was studied by examining the samples using SDS-PAGE, western blot and Bradford methods.

### Western blot analysis

Samples were loaded on 12% SDS-PAGE for analysing about western blotting method. After running the gel, proteins were electroblotted on a presoaked nitrocellulose membrane (Merck-India, #WHA7191014) using the transfer buffer including 25mM Tris, 192mM glycine and 6.20% v/v methanol (pH 8.3) and run on 400 mA for 2.5 h. The membrane was presoaked with 1× PBST [NaCl 3.0 g, KH2PO4 0.2 g, NaHPO4 1.15 g and KCl 0.2 g/l (pH 7.4) containing 0.5% Tween-20], and blocking was done with blocking buffer [5% skimmed milk powder in 1× PBS (NaCl 3.0 g, KH2PO4 0.2 g, NaHPO4 1.15 g and KCl 0.2 g/l, pH 7.4)] and incubated for overnight at 4 °C. The blocked membrane was washed with 1× PBS three times for 15 min each. Anti-HN antibody (Abcam, USA, #ab226322) at a dilution of 1:500 specific to the target protein was incubated with the membrane at room temperature for 2 h. After washing of unbound primary antibody with 1× PBST, the secondary antibody anti-mouse IgG peroxidase conjugate HRP (#GR129315-1) at 1:1000 dilution was added at room temperature for 2 h. For protein detecting, 3,3′,5,5′ tetramethylbenzidine (TMB) (Invitrogen) was used as a substrate, and 1M H_2_SO_4_ stopped the reaction.

### Purification

*B. subtilis WB800* carrying pHT43-HN recombinant plasmids was cultivated overnight in 250 ml of LB broth containing chloramphenicol, and the expression was induced with 1 mM of IPTG, followed by incubation for 24 h. When the incubation ended, the crude culture broth was centrifuged at 10,000 rpm at 4 °C for 10 min, and the supernatant containing the HN protein was recovered. The purification of the recombinant HN protein was performed in three steps. First, a total of 200 ml of culture supernatant was freeze-dried, decreased the volume to 10 ml with a freeze dryer and then was dialyze with Tris-Cl (pH 8.5). The collected supernatant was precipitated according to the method discussed in the section expression, and it was subsequently suspended in 20 ml of sodium phosphate buffer (pH 6.0). For the 63-kDa HN protein purification, a Q Sepharose® column (2 × 25 cm) was used. Then, 20 ml of the protein suspension was gently and carefully transferred to the column, and the washing process was performed according to the standard method. To wash the proteins attached to the substrate, a concentration gradient of 0 to 1 mM of NaCl was used. The obtained product was then loaded onto a 20-ml Ni-NTA Agarose column (Qiagen). Afterwards, the column was washed and then eluted with a gradient of 1 to 320 mM of imidazole. Finally, all the samples with peak fractions were stored at 4 °C for further analysis. All purification steps were carried out at 4 °C [9].

### Characterization of the recombinant hemagglutinin–neuraminidase

#### Neuraminidase enzymatic activity assay

The neuraminidase activity measurement of the recombinant HN protein at 37 °C in white 96-well plates with a total volume of 150 μl was performed using the Neuraminidase Activity Measurement Kit (Abcam- USA, # ab138888) according to the manufacturer’s instructions. The fluorescence level was monitored every minute for 30 min using a GEMINI spectrofluorometer with excitation and emission wavelengths of 320 and 450 nm, respectively.

#### Salt and pH dependence of neuraminidase activity

The neuraminidase activity of the recombinant HN protein was performed in buffers with different pH values at a final concentration of 50 mM, containing 4 mM of CaCl2, 5–160 mM of NaCl and 50 μM of the Neuro Blue substrate (Sigma, USA, #1911). Two buffering systems were used in this study, i.e. NaOAc for pH values of 4.0–7.0, and MES for pH values of 5.0–7.0. All reactions were conducted at 37 °C in white 96-well plates in a total volume of 150 ml.

#### Statistical analysis

The serological data analysed using ANOVA one-way data for challenge assays were compared using Pearson’s chi-squared test. Moreover, *P* < 0.05 was considered as the threshold for significant differences.

## Results

### Amplification and characterization of the HN gene

**Fig. 1:**
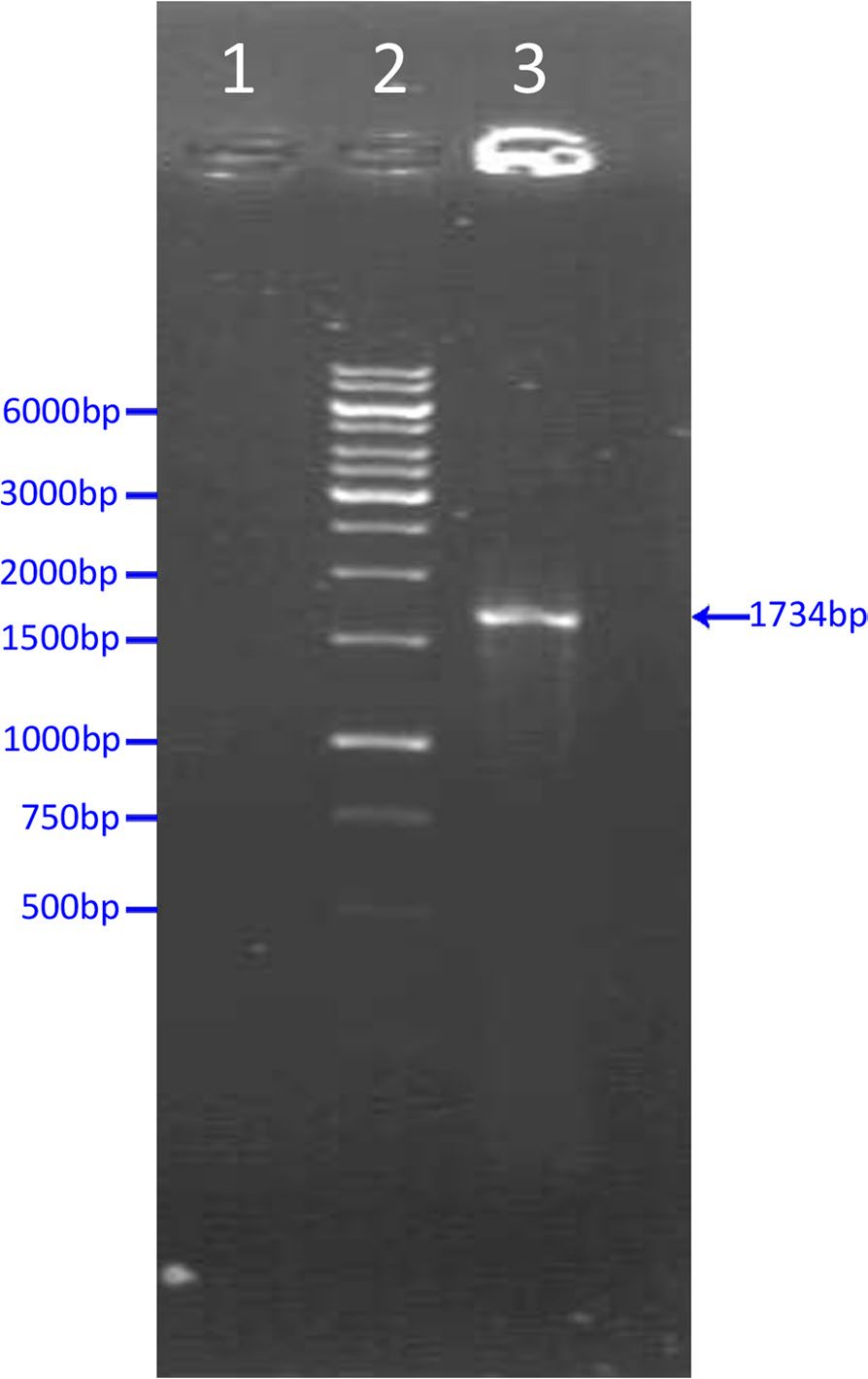
Gel electrophoresis analysis of HN gene (1734 bp) RT-PCR products. Lane 1: negative control. Lane 2: 1-kb DNA size marker (Fermentas #SM0311). Lane 3: demonstrating a thick bond of 1734 bp amplicon

**Fig. 2:**
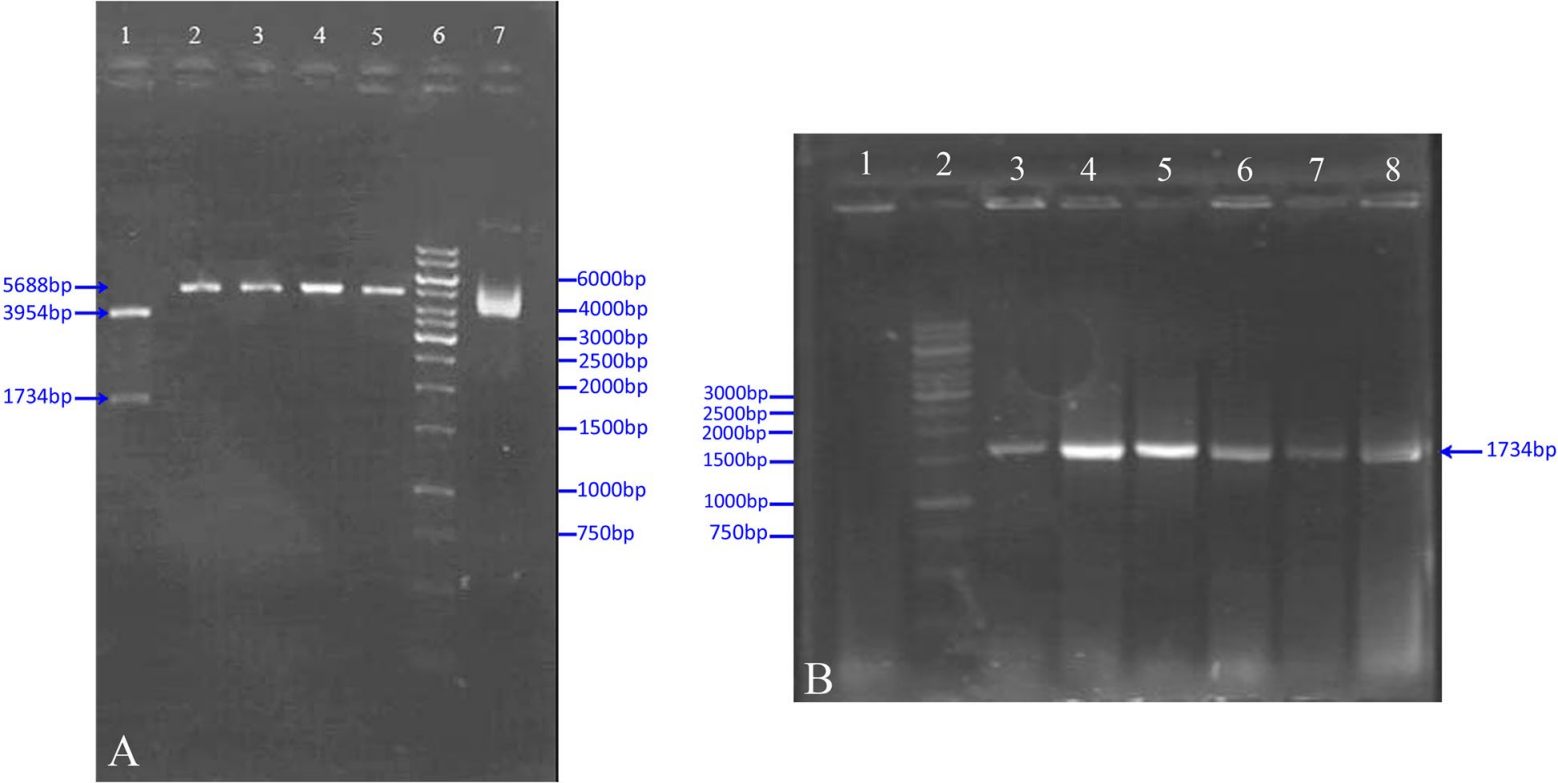
Enzymatic digestion map of the new pGet-HN recombinant T/A cloning vector. **A** Lane 1: BamHI/Xb double digestion on the HN-containing recombinant T/A cloning vector (clone) revealed expected 3954-bp and 1734-bp fragments. Lanes 2–5: single digested, revealed expected fragment (5688 bp). Lane 6: 1-kb DNA size marker (Fermentas #SM0311). Lane 7: undigested plasmid of the new HN-containing recombinant T/A cloning vector. **B** HN, gene PCR with specific primer, six white colonies were selected randomly. Lane 1: negative control (blank). Lane 2: 1-kb DNA size marker (Fermentas #SM0311). Lanes 3–8: HN-gene fragment (1734 bp)

**Fig. 3:**
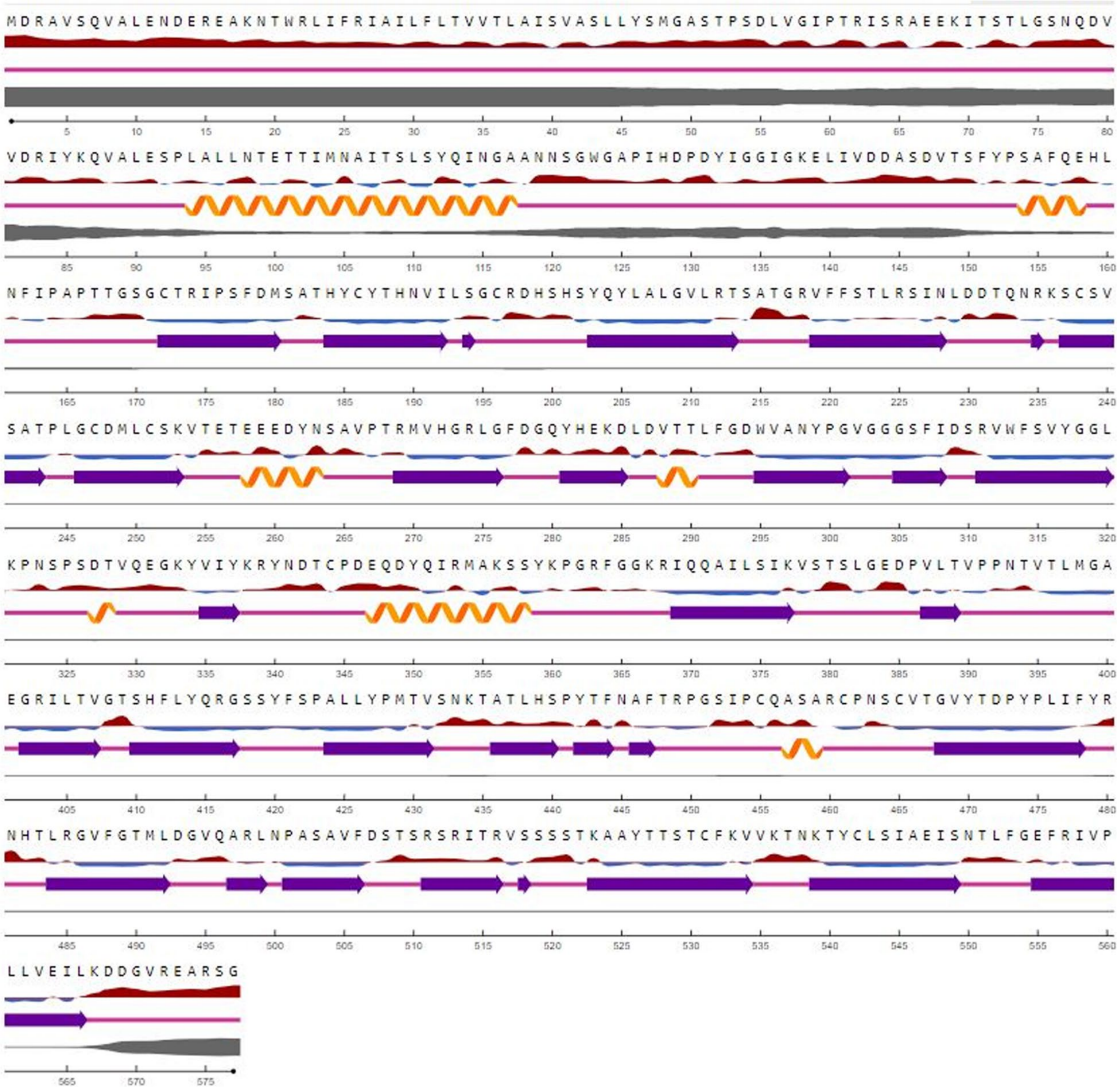
Deduced amino acid sequence and the secondary structure of the HN protein. The helices are shown by a yellow colour, the strands are represented as purple arrows and the coils are represented as pink lines

The full-length sequence of the HN gene (1734 bp) was amplified by RT-PCR using the specifically designed primers (Fig. 1). The produced fragment after extraction from agarose gel was cloned into the T/A cloning vector, and it was confirmed using PCR (Fig. 2a), and the analysis of enzyme digestion was verified (Fig. 2b). Sequencing validated the correctness of the HN gene ORF in the T/A cloning vector. Chromas was used to analyse the sequence (version 1.45, Australia). After determining the recombinant vector sequence, the blast analysis showed that the sequence belonged to a new virus generation with a 98% similarity to the HN gene of the Newcastle virus. Therefore, due to the lack of similarity of 100%, this sequence was taken as the IRI1399 isolate, and it was registered in the gene bank with the ID number MT551214.1 as the sequence native to Iran. Following that, the HN segment was sub-cloned into the pHT43 donor plasmid, as previously reported in the “Methods” section. PCR and enzymatic digestion were used to validate the recombinant vector (results not presented). The analysis of the nucleotide sequence of HN revealed an ORF of 1734 bp, encoding a protein with 577 amino acids. The molecular mass of the recombinant protein was predicted to be 63 kDa with a theoretical pI of 7.13 using the ExPASy server (https://web.expasy.org/cgi-bin/protparam/protparam). It was also observed that the total numbers of negative and positive charge residues were equal (total number of negatively and positively charged residues, 53). Moreover, evaluating the protein’s secondary and 3D structures showed that the secondary structure of the HN protein consisted of three domains, seven alpha-helix regions (19.3%), 31 extended strand regions (18.21%) and 39 random coil regions (58.6%) (Fig. 3).

**Fig. 4:**
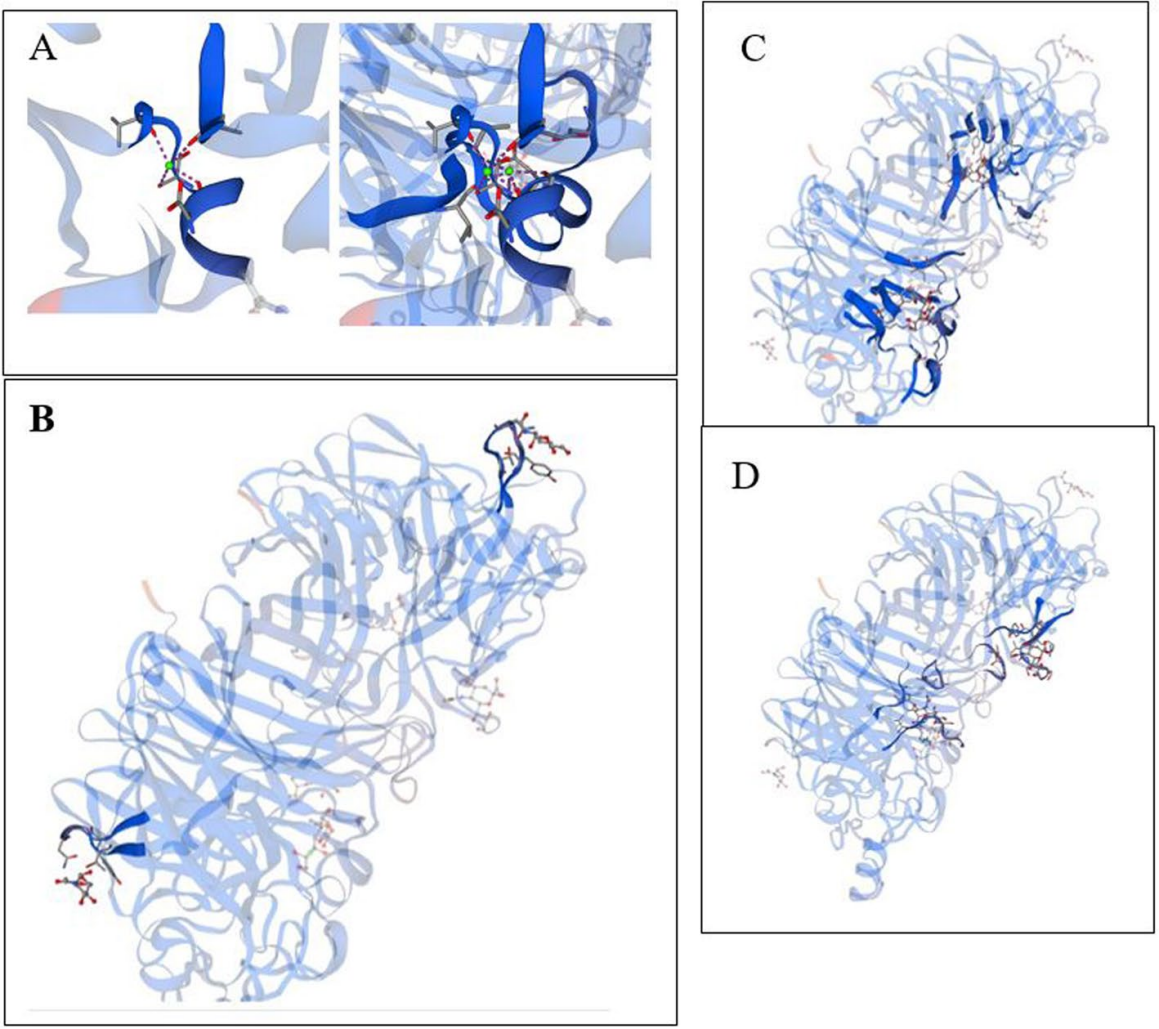
Evaluation of the protein structure in terms of calcium ion position, 2-deoxy-2,3-dehydro-*N*-acetyl-neuraminic acid and sugar. **A** Residuesof calcium ions, atomic arrangement and interaction. **B** Residuesof 2-deoxy-2,3-dehydro-*N*-acetyl-neuraminic acid, atomic arrangement and interaction. **C** Residuesof 2-(acetylamino)-2-deoxy-*A*-d-glucopyranose, atomic arrangement and interaction. **D** Residuesof O-sialic acid, atomic arrangement and interaction

**Fig. 5:**
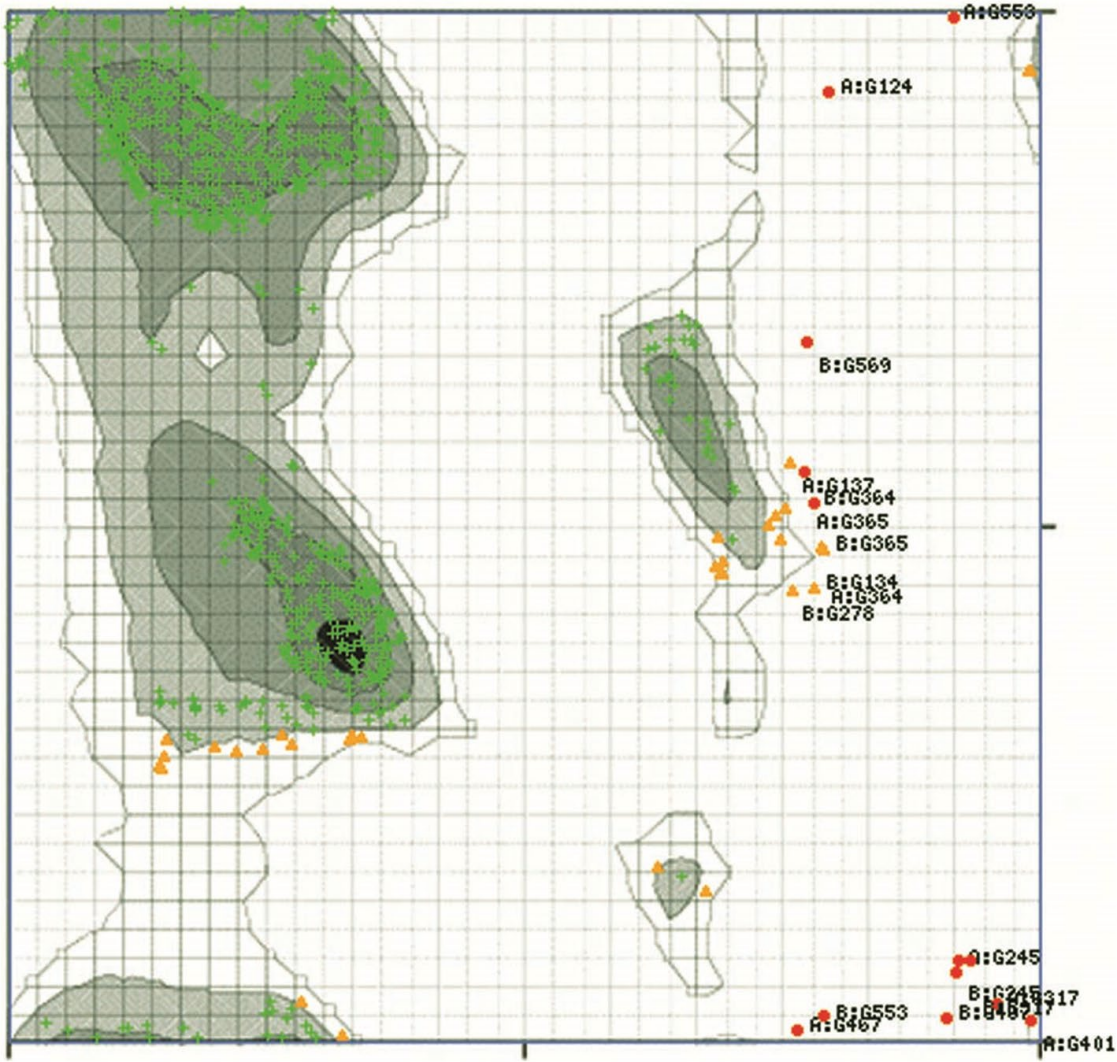
The Ramachandran plot of the refine model (the software MolProbity: http://kinemage.biochem.duke.edu)

The prediction of the 3D structure of the recombinant HN protein was performed and compared to the native HN protein using Swiss modelling. The recombinant HN protein-predicted structure showed high identity (46.3% *E*-value 0.00e−1) by homology with the HN protein (SV5:PDB: 1Z4V). The structure of the recombinant HN protein was similar to that of paramyxoviruses [25], indicating that the recombinant HN protein and native HN protein both had two calcium ion regions, i.e. CA.4 five residues within 4Å (E.256, D.261, S.264, V.266, V.296) and five PLIP interactions (A:D.261, A:S.264, A:S.264, A:V.266, A:V.296) and CA.9, four residues within 4Å (D.261, S.264, V266, V.269) and four PLIP interactions (B:D.261, B:S.264, B:V.266, B:V.296) (Fig. 4a); two 2-deoxy-2,3-dehydro-*N*-acetyl-neuraminic acids, including DAN.2 (11 residues within 4Å (R.174, I.175, E.258, Y.299, Y.317, R.363, E.401, R.416, V.466, R.498, Y.526) and nine PLIP interactions (hydrophobic interactions: A:Y.299-hydrogen bonds: A:Y.317, A:Y.317, A:R.363, A:E.401, A:R.416-salt bridges: A:R.174, A:R.416, A:R.498) (Fig. 4b); two sugars (2-(Acetylamino)-2-deoxy-*A*-d-glucopyranose), including NDG.3 and NDG.7, each with three residues and three PLIP interactions (Fig. 4c), and finally, two sugars (*O*-sialic acid) with nine residues and ten PLIP interactions (Fig. 4d). The minimum energy calculation in the Swiss-PdbViewer shows that the protein has a stable structure. The Ramachandran plot of the refined model estimated the number of residues in the favoured region to be 453 (92.4%), the number of residues in the allowed region to be 25 (5.1%), and the number of residues in the outlier region to be 12 (2.4%) (Fig. 5).

### Expression and purification of the recombinant protein

**Fig. 6:**
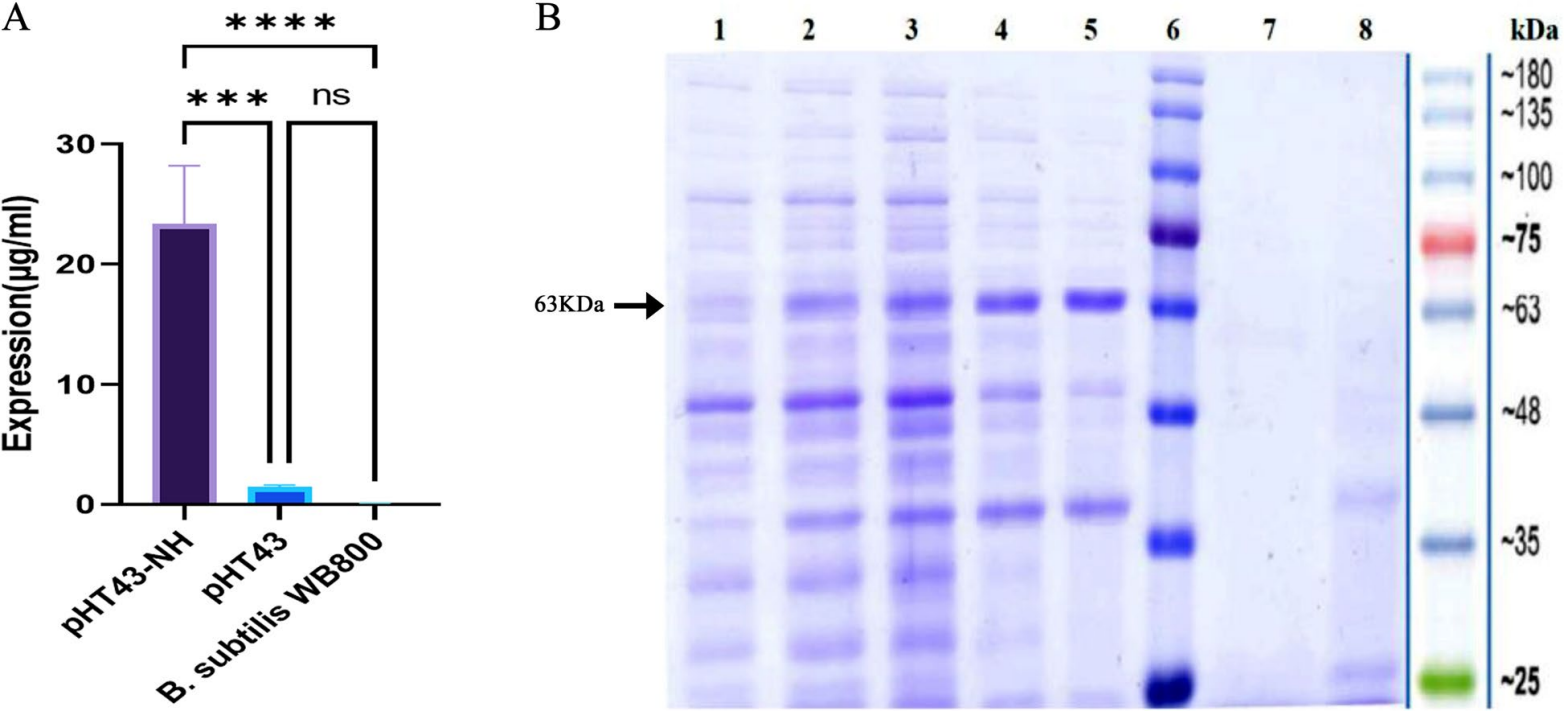
Evaluation of recombinant protein expression in *B. subtilis*. **A** Summary of the normalized quantities of expression proteins in three bacterial groups. One-way analysis of variance (ANOVA) by Tukey’s method showed that in level *P* ≤ 0.05, the observed difference in pHT43_NH expression IPTG-induced pHT43 and the *B. subtilis* WB600 groups is statistically significant, whereas there was no statistically significant relationship between pHT43 and *B.subtilis* WB600. **B** Investigation of expression and secretion of recombinant HN protein (63KDa) in culture medium with acrylamide gel 12%. Column 1: expression before induction. Columns 2 to 5: gradual increase in the expression from 1 to 8 h after induction. Column 6: molecular weight (#SL7001-Sinaclon, Iran). Column 7: lack of protein secretion in the culture medium before induction. Column 8: lack of recombinant protein secretion in bacterial cells containing plasmid without HN genes

**Fig. 7:**
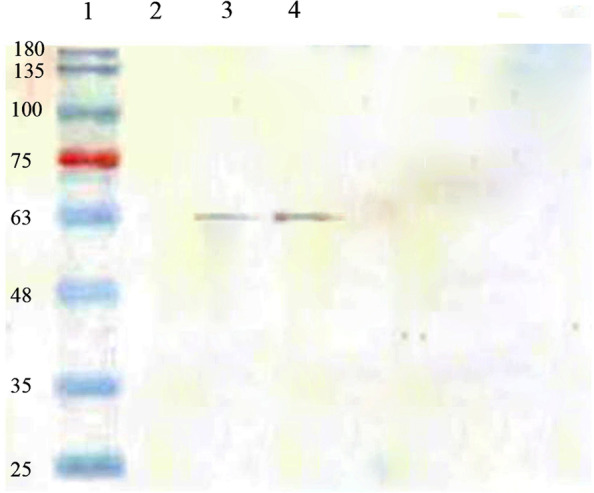
The result of western blotting to increase the sensitivity in the detection of membrane proteins. Lane 1: marker weight (#SL7001-Sinaclon, Iran). Lane 2: pre-induced protein without expression. Lane 3: a positive control sample, a positive western bloat response to a His-Tag protein sample weighing 63 kD. Lane 4: confirmation of recombinant HN protein (63KD) expression, 8 h after induction. Lane 5: lack of recombinant protein secretion in bacterial cells containing plasmid without HN genes

Investigation of the expression in three bacterial groups (i.e. A, B and C) tested with the sample before induction was performed using the Bradford method and then on polyacrylamide gel (Fig. 6a, b). The SDS-PAGE results showed that the expression of the recombinant HN protein gradually increased up to 8 h after induction (63-kDa expressed protein in *Bacillus subtilis*). The results also showed that the expressed protein band in *Bacillus subtilis* had a weight of approximately 63 kDa in western blotting (results for groups B and C are not presented). The HN protein confirmation test results were analysed using the western blot analysis with mouse monoclonal antibody, i.e. the anti-HN in Fig. 7, which confirms the expression of the HN protein (Fig. 7).

**Fig. 8:**
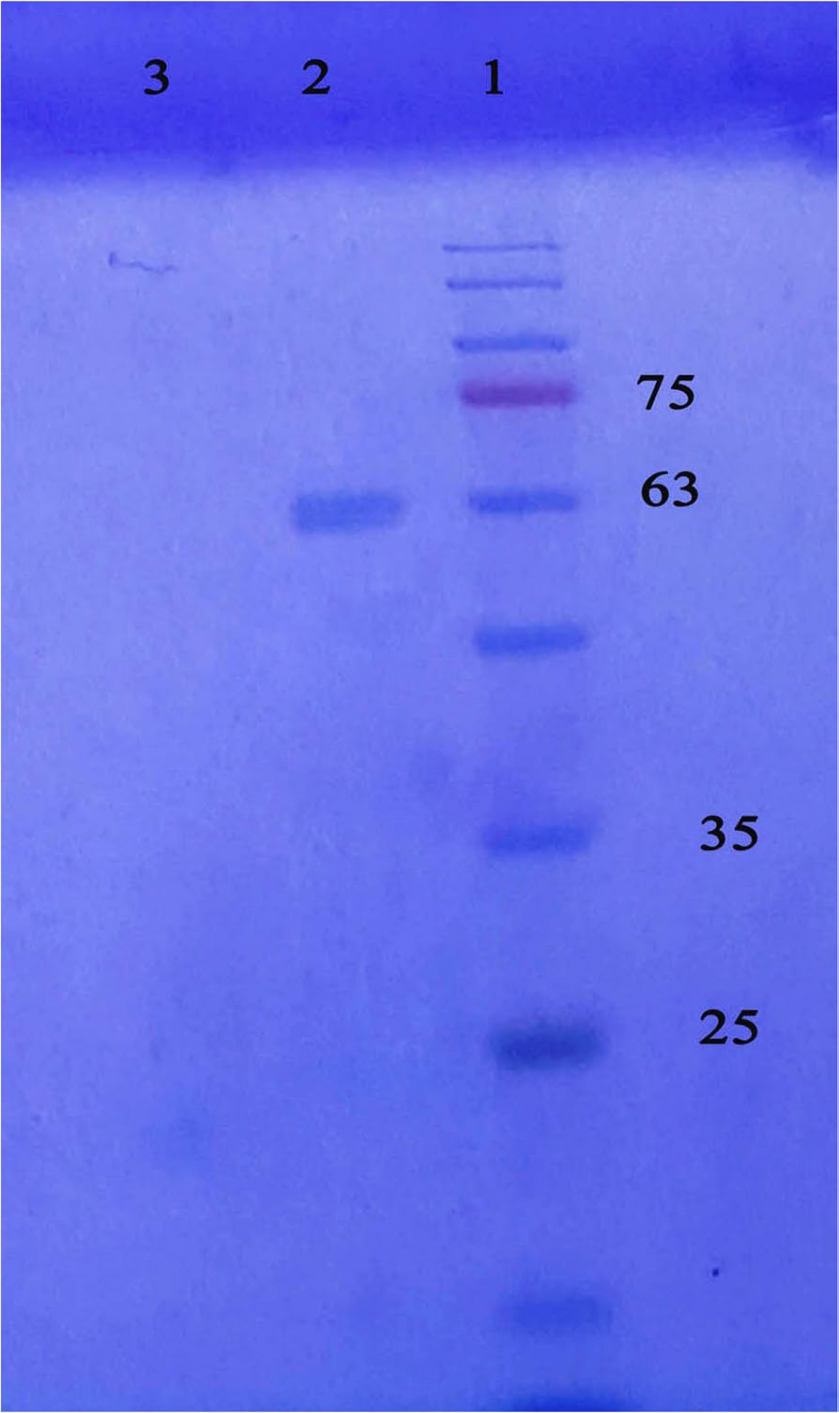
The HN protein was purified by chromatography. Lane 1: marker weight (#SL7001-Sinaclon, Iran). Lane 2: purified HN protein (63 kD). Lane 3: negative control (culture medium)

A three-step purification protocol was employed for the recombinant HN purification. After dialysis, the cultural supernatant was loaded onto a Q sepharose cation-exchange column under an acidic pH of 4.6, as described in the “Purification” section. The pooled eluted fractions containing neuraminidase were purified through chromatography on a Ni-NTA affinity column. SDS-PAGE analysis revealed a single band with a molecular weight of 63 kDa.The study of neuraminidase specific activity suggested that this double-step chromatography would cleanse HN by about 3.8 fold (detailed description of the calculation is given in Table 1 (Fig. 8)).

**Table 1:** Purification of recombined HN protein

Fraction	Volume (ml)	Protein (mg)	Activity neuraminidase (unit)	Specific activity (pmol/min/mg)	Fold purification	Yield %
Cultural supernatant	200	40.6	63.34	1.5	1×	100
Pooled eluates from SP	20	29.15	62.66	2.14	1.4×	98
Pooled eluates from Ni-NTA	20	10.64	61.84	5.8	3.8×	97

*Specific activity = (activity enzyme/protein)

**Fold purification = (specific activity/starting special activity)

***Yield = [(activity enzyme/starting activity enzyme) × 100]

### pH and chloride dependence of the recombinant neuraminidase activity

**Fig. 9:**
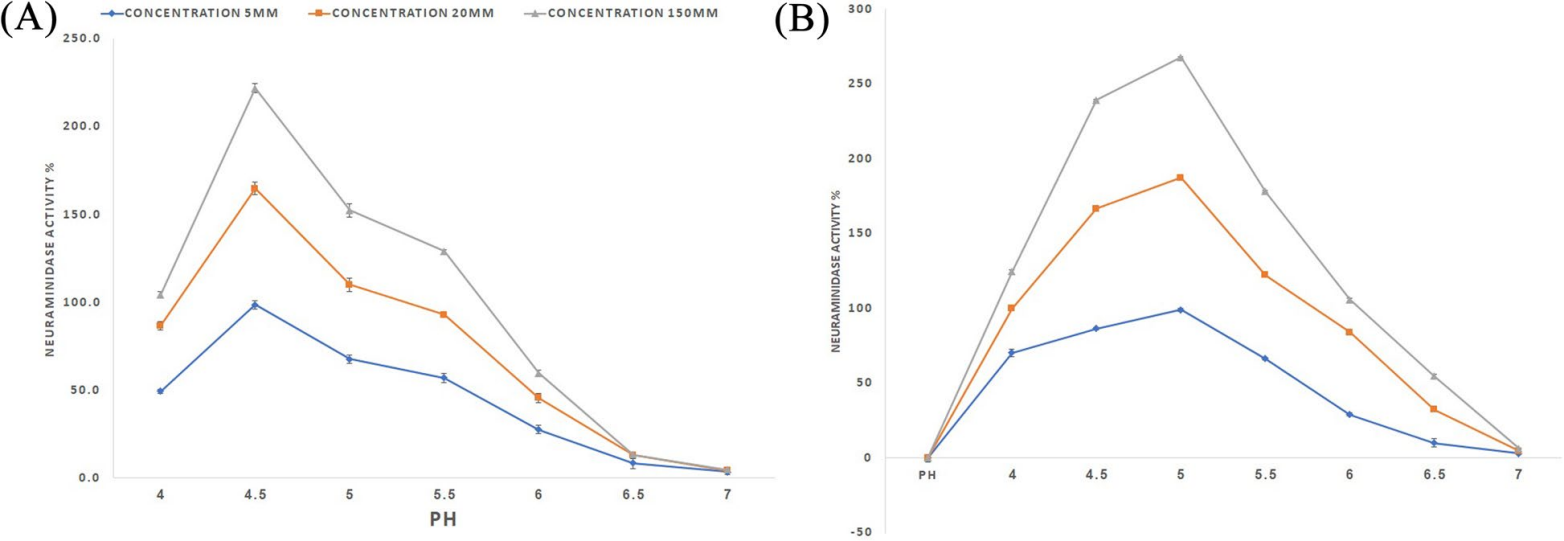
Neuraminidase activity of HN protein is pH and chloride-dependent. **A** Activity of purified recombinant HN protein in the presence of 5 (“”, •blue line), 20 (“■”, orange line) and 150 (“◄”, grey line) mMNaCl. **B** Activity of native HN from viral lysate in the presence of 10 (“•”, blue line), 90 (“■”, orange line) and 160 (“◄”, grey line) mMNaCl

Paramixovirus neuraminidase activity had been shown to be pH-dependent and chloride ion-dependent. Paramyxoviruses prefer acidic pH and low chloride ion concentrations, suggesting that paramyxovirus neuraminidase may act in some cellular components, such as the ER or Golgi apparatus, to prevent virions from accumulating before being released from host cells [35]. Comparing the affect of chloride ion and pH on recombinant purified HN protein, neuraminidase showed interest among local viral HN protein in viral lysate. The neuraminidase activity of native HN protein showed an optimal protein activity at a pH of 5. Increasing chloride ion concentration from 5 to 160 mM was decreased 10% neuraminidase activity. A comparison of the neuraminidase activity of recombinant purified HN protein displayed an optimum pH of 4 and decreased 35% neuraminidase activity upon increasing chloride ion concentration from 5 to 160 mM. Our observations showed that both native HN protein and recombinant HN protein prefer an acidic environment, and their activity is reduced in high chloride buffers (Fig 9).

## Discussion

The paramyxovirus HN protein is a type II glycoprotein anchored in the viral envelope membrane connected to the membrane by a hydrophobic domain [3, 9, 35]. HN protein, along with protein F, is a major factor in viral virulence. In other words, this surface protein can be an important target for the host’s immune response to fight the virus [36]. Choosing the right host to express and produce recombinant protein is one of the most major issues. Using bacillus expression vectors for eukaryotic gene expression has two advantages: easy purification and protein secretion into the culture medium. However, this expression system cannot implement modifications after translating eukaryotic proteins [37]; however, since many eukaryotic proteins retain their three-dimensional structure and complete biological activity in the non-glycosylated form, they can be expressed in *Bacillus subtilis* [38–41].This study successfully used the *Bacillus* expression system to express the HN protein of the Newcastle disease virus that has a biologically active form. The main limitation of this system is the production of extracellular proteins that destroy foreign recombinant proteins. This problem has been solved using engineered host strains such as WB600 and WB800 [42, 43]. In the present study, the HN protein of the Newcastle disease virus was expressed in a genetically modified strain of *Bacillus subtilis* called WB800 that has higher competency to uptake foreign DNA molecules. The WB800 is lacking six extracellular proteases and hence suited for extracellular recombinant protein synthesis [44, 45]. In the current study, the HN gene of the Newcastle disease virus was successfully cloned and expressed in *B. subtilis* strains WB800, and 10 μg per 200 ml culture of recombinant HN protein was produced successfully. pHT43-HN expression vectors contained the Pgrac strong promoter, and signal peptide sequence of the amy-Q (𝛼 amylase) gene of *Bacillus amyloliquefaciens* has been reported for the efficient secretion of recombinant proteins through the Sec pathway [45, 46]. Proteins generated as pre-protein complex signal peptides translocate to the cell membrane and bind to the secretory translocase complex identified by the Sec-dependent secretory pathway signal peptide. After then, protein is transferred out of the cell a particular signal peptidase removes the signal peptide at the cleavage site [43]. Thus, the selection of suitable signal peptide affects the rate and yield of the secreted protein. One option for selecting the signal peptide is using commercially available signal peptides, literature survey and review of the proteome of the host organism for signal peptide. The latter is associated with the production of homologous secreted proteins [43]. In actuality, in the absence of a signal peptide, any protein generated in the absence of a signal peptide is kept in the cytoplasm [8, 43, 47]. Proteins formed as pre-protein complexes with N-terminal signal, peptidescross the cell membrane through the Sec associated secretory pathway. It interacts with the signal peptide-recognized secretory translocase complex. After the signal peptide is removed at the cleavage site by a unique signal peptidase, the protein is transported out of the cell [8].Therefore, choosing the suitable signal peptide affects the rate of protein secretion and, ultimately, the yield of the secreted protein [40, 47, 48]. The expression vector used in this study included the -amy signal peptide, which has a high propensity for heterogeneous protein secretion. In this study, an expression vector containing the *amy-Q* signal peptide was used, which has a high potential for heterogeneous protein secretion. There have also been several reports of using this signal peptide to express human IL3 and INF [49, 50]. Westers et al. had previously tested various signal peptides and promoters for IL-3 synthesis [51]. Accordingly, the best combination was using *amy-Q* signal peptide with pHT43 promoter, resulting in maximal IL-3 protein production and close to zero cytoplasmic retention [51]. These results confirm our choice of an expression vector containing the *amy-Q* signal peptide. WB800 cells have a high potential for recombinant protein secretion. However, previous data reports showed that plasmid instability is not a rare phenomenon in *B. subtilis* [52, 53]. Plasmid instability is widespread in low-copier plasmids [54]. However, in the present study, the plasmid used has good stability in the host cell due to the contained *ColE1* origin of replication associated with the relaxed type of replication control [41]. Another significant drawback of the bacillus secretory mechanism is extracellular proteases [44, 55]. Six extracellular proteins are in the WB800 strain, making it an ideal host for secretory proteins. Previous studies on the recombinant production of eukaryotic proteins by *B. subtilis* have reported varying amounts of secretion from 100 micrograms to 1000 mg [44, 51, 56]. HN recombination levels were high. During the three-step filtration, the final purified HN yield of approximately 10 mg/L *B. subtilis* cell culture contained a recombinant plasmid. The paramyxovirus activity of neuraminidase depends on the pH and the concentration of chloride ions. It prefers acidic and low pH. The concentrations of chloride ions indicate that paramyxovirus neuraminidase functions in certain cellular compartments, such as the ER or Golgi [57]. In the current study, we showed that native neuraminidase and recombinant neuraminidase are active better in acidic environments, and their activities decrease with increasing chloride ion concentration.

## Conclusions

In this study, a successful expression and purification of functional HN of NDV in the *B. subtilis* system are described. Sequencing the HN gene indicated that it consisted of 1734 bp, encoding a protein of 544aa. Through precipitation, gel filtration and anion exchange, the recombinant protein was purified to 3.8-fold with a specific activity of 5.8 U/mg. The purified enzyme was homogenous on SDS-PAGE, and its molecular weight was estimated to be 63 kDa. The recombinant HN protein started its optimum activity at pH 5.0, and increasing chloride ion concentration from 5 to 160 mM decreased 10% neuraminidase activity. Although the post-translational processes such as glycosylation do not occur in the *B. subtilis* expression system, the recombinant HN protein retains the neuraminidase activity properties similar to the native protein. *Bacillus subtilis* (WB800) expression system which has desirable features such as unbiased codon usage [58], of most extracellular proteases [44] and endotoxin, has a high capacity to secrete a soluble and active form of protein into the culture medium and prevent the formation of inclusion bodies [59, 60]. It provides a good platform for producing recombinant HN protein, and it retained the neuraminidase activity with characteristics similar to those of native HN protein. The establishment of this expression and purification system has allowed us to explore the biochemical characteristics of paramyxovirus HN further obtain material that can be suggested as a new generation of NDV vaccine candidate.

## Data Availability

All data generated or analysed during this study are included in this published article [and its supplementary information files].

## Abbreviations

amyQ: 𝛼-Amylase
*B. subtilis*: *Bacillus subtilis*
*E. coli*: *Escherichia coli*
IPTG: Isopropyl 𝛽-d-1-thiogalactopyranoside
IL-3: Interleukin 3
INF: Interferon
SDS-PAGE: Sodium dodecyl sulfate polyacrylamide gel electrophoresis
HN: Hemagglutinin–neuraminidase
NP: Nucleoprotein
P: Phosphoprotein
M: Matrix
F: Fusion
NDV: Newcastle disease virus
ND: Newcastle disease
ORF: Open reading frames
pI: Isoelectric point
RNA: Ribonucleic acid
BLAST: Basic Local Alignment Search Tool
